# Indicators of Abdominal Adiposity and Carotid Intima-Media Thickness: Results from the Longitudinal Study of Adult Health (ELSA-Brazil)

**DOI:** 10.5935/abc.20180273

**Published:** 2019-03

**Authors:** Michaela Eickemberg, Leila Denise Alves Ferreira Amorim, Maria da Conceição Chagas de Almeida, Estela Maria Leão de Aquino, Maria de Jesus Mendes da Fonseca, Itamar de Souza Santos, Dora Chor, Maria de Fátima Sander Diniz, Sandhi Maria Barreto, Sheila Maria Alvim de Matos

**Affiliations:** 1 Universidade Federal da Bahia, Salvador, BA - Brazil; 2 Escola Bahiana de Medicina e Saúde Pública, Salvador, BA - Brazil; 3 Centro de Pesquisas Gonçalo Moniz, Fundação Oswaldo Cruz (FIOCRUZ), Salvador, BA - Brazil; 4 Escola Nacional de Saúde Pública - Fiocruz, Rio de Janeiro, RJ - Brazil; 5 Universidade de São Paulo, São Paulo, SP - Brazil; 6 Universidade Federal de Minas Gerais, Belo Horizonte, MG - Brazil

**Keywords:** Cardiovascular Diseases, Risk Factors, Metabolism, Metabolic Syndrome, Abdominal Obesity, Atherosclerosis, Carotid Intima-Media Thickness

## Abstract

**Background:**

Abdominal adiposity is a risk factor for cardiovascular disease.

**Objective:**

To determine the magnitude of the association between abdominal adiposity, according to five different indicators, and the carotid intima-media thickness (CIMT).

**Methods:**

Data from 8,449 participants aged 35 to 74 years from the ELSA-Brazil study were used. The effect of waist circumference (WC), waist-to-hip ratio (WHR), conicity index (C index), lipid accumulation product (LAP) and visceral adiposity index (VAI) on CIMT were evaluated. Data were stratified by gender and analyzed using multivariate linear and logistic regressions. A significance level of 5% was considered.

**Results:**

Participants with CIMT > P75 showed a higher frequency of abdominal adiposity (men >72% and women >66%) compared to those with CIMT < P75. Abdominal adiposity was associated with the mean CIMT, mainly through WC in men (0.04; 95%CI: 0.033; 0.058). The abdominal adiposity identified by the WC, WHR, LAP, and VAI indicators in women showed an effect of 0.02 mm on the CIMT (WC: 0.025, 95%CI: 0.016, 0.035; WHR: 0.026, 95%CI: 0.016, 0.035; LAP: 0.024, 95%CI: 0.014; 0.034; VAI: 0.020, 95%CI: 0.010, 0.031). In the multiple logistic regression, the abdominal adiposity diagnosed by WC showed an important effect on the CIMT in both genders (men: OR = 1.47, 95%CI: 1.22-1.77, women: OR = 1.38; 95%CI: 1.17-1.64).

**Conclusion:**

Abdominal adiposity, identified through WC, WHR, LAP, and VAI, was associated with CIMT in both genders, mainly for the traditional anthropometric indicator, WC.

## Introduction

Abdominal obesity is a traditional risk factor for cardiovascular diseases.^^1^^ In Brazil, the prevalence of abdominal obesity, estimated by the National Health Survey *(Pesquisa Nacional de Saúde),* according to the cut-off points for waist circumference (WC) of the World Health Organization,^^2^^ was 52.1% for women and 21.8% for men in 2013.^^3^^

Several mechanisms have attempted to explain how abdominal adiposity becomes a risk factor for cardiovascular disease. It is a consensus that abdominal adipose tissue has complex metabolic functions and produces numerous mediators that trigger specific, dynamic and inflammatory reactions.^^4^^

Atherosclerotic lesions increase the risk for cardiovascular diseases. The carotid intima-media thickness (CIMT) is a marker of subclinical atherosclerosis and a predictor of myocardial infarction and cerebrovascular accident.^^5^^ The association between abdominal adiposity and subclinical atherosclerosis has been documented in different populations.^^6^^^,^^^7^^ However, even though the CIMT is associated with abdominal adiposity, it is yet to be fully established how much this adiposity, measured by different clinical and other unusual indicators, is associated with subclinical atherosclerosis.

Studies have suggested that WC, waist-to-hip ratio (WHR) and visceral adiposity index (VAI) may predict subclinical atherosclerosis.^^6^^^,^^^8^^^,^^^9^^ Most studies on this subject were performed in Europe, Asia and the United States, and use the WC and WHR to define abdominal adiposity and its association with cardiovascular diseases. Indicators that provide indirect information on lipid overaccumulation and visceral fat function associated with cardiovascular events, such as VAI^^10^^ and the lipid accumulation product (LAP)^^11^^, need to be further explored. The conicity index (C index) stands out as a discriminator of high coronary risk in Brazilian studies, especially when a black population is being investigated.^^12^^ On the other hand, there are no studies that investigated the effect of adiposity diagnosed by this index on CIMT.

The aim of this study was to determine the magnitude of the association between abdominal adiposity, according to different diagnostic indicators (WC, WHR, C Index), and between indexes that reflect visceral adipose tissue dysfunction (LAP and VAI) and CIMT among the participants of ELSA-Brazil.

## Methods

### Study design and population

The ELSA-Brasil study included in its baseline 15,105 civil servants, aged 35-74 years, connected to six teaching and research institutions in three Brazilian regions (South, Southeast and Northeast). More details on the study methodology can be found in an earlier publication.^^13^^

Interviews and collection of anthropometric and biochemical measurements were carried out by a trained and certified team. A more detailed publication is available on the standardization and quality assurance procedures and the quality of uniformization regarding the conducts adopted in the ELSA-Brazil.^^14^^

### Exclusion Criteria

**Figure 1:**
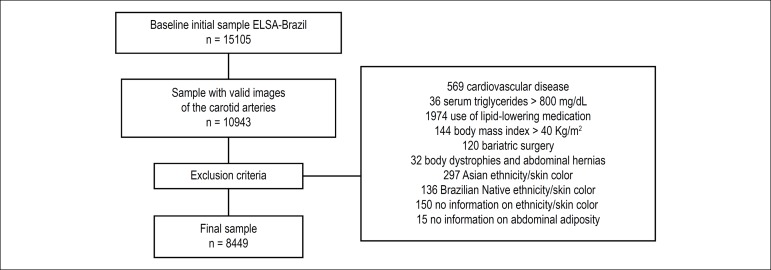
Study sample selection flowchart. Note: Percentage of exclusion (sample with valid images and final sample): 23%.

In order to keep a healthy sample and to avoid biases related to CIMT, of the 10,943 participants with a valid image for both common carotid arteries, we excluded 569 patients who declared having cardiovascular disease, 36 with serum triglycerides > 800 mg/dL, 1,974 patients using lipid-lowering medication, 144 with BMI > 40 kg/m^2^ and 120 who underwent bariatric surgery. To avoid biases related to abdominal fat measurement, 32 participants with body dystrophies and abdominal hernias were excluded. We also excluded the participants who self-declared as having Asian and Native Brazilian ethnicity/skin color due to the small number (297 and 136, respectively), 150 participants who did not declare ethnicity/ skin color and 15 without information on indicators of abdominal adiposity. The final sample consisted of 8,449 participants (Figure 1). Some participants had more than one condition for exclusion.

### Carotid intima-media thickness (CIMT)

All the research centers collected the CIMT measurement using a standardized method, utilizing an Aplio XG(tm), Toshiba equipment, with a 7.5 MHz linear transducer. The technique used in the study has been published elsewhere.^^15^^^,^^^16^^ For this article, CIMT was defined as the mean of the mean values of the right and left carotid arteries. The 75^th^ percentile was used to dichotomize this variable according to gender (male: 0.69 mm, female: 0.64 mm). The 75^th^ percentile was based on technical consensuses and previous studies.^^17^^

### Indicators of abdominal adiposity

Anthropometric measurements were obtained using standardized equipment and techniques. The WC was measured at midpoint between the inferior border of the costal arch and the iliac crest, at the median axillary line and at the hip circumference at the maximal protrusion of the gluteal muscles, over the trousers of the study clothing. These circumferences were used to calculate the WHR. The C index was calculated using the formula: $\mathrm{WC}\left(m\right)/0.109\;x\;\sqrt{\mathrm{Weight}}\left(\mathrm{kg}\right)/\mathrm{Height}\left(m\right)$.^^18^^

The LAP^^19^^ was calculated using gender-specific equations: $\mathrm{Men}:\;\mathrm{WC}\left(\mathrm{cm}\right)-65\;x\;\mathrm{triglycerides}\left(\mathrm{mmol}/L\right);\;\mathrm{Women}:\;\mathrm{WC}\left(\mathrm{cm}\right)-58\;x\;\mathrm{triglycerides}\left(\mathrm{mmol}/L\right)$, as well as the VAI:^^19^^ $\mathrm{Men}:\;\left(\mathrm{WC}\left(\mathrm{cm}\right)/39.68+\left(1.88\;x\;\mathrm{body}\;\mathrm{mass}\;\mathrm{index}\left(\mathrm{kg}/m^{2}\right)\right)\right)\;x\;\left(\mathrm{triglycerides}\left(\mathrm{mmol}/L\right)/0.81\right)\;x\;\left(1.31/\mathrm{HDL}\;\mathrm{cholesterol}\left(\mathrm{mmol}/L\right)\right);\;\mathrm{Women}\;\left(\mathrm{WC}\left(\mathrm{cm}\right)/36.58+1.89\;x\;\mathrm{body}\;\mathrm{mass}\;\mathrm{index}\left(\mathrm{kg}/m^{2}\right)\right)\;x\;\left(\mathrm{triglycerides}\left(\mathrm{mmol}/L\right)/0.81\right)\;x\;\left(1.52/\mathrm{HDL}\;\mathrm{cholesterol}\left(\mathrm{mmol}/L\right)\right)$.

The indicators were categorized in the presence and absence of abdominal adiposity, according to the cut-off points defined by Eickemberg et al.,^^20^^ Respectively, the following values were used for white, brown and black individuals: WC: men 89.9 cm; 90.2 cm and 91.7 cm; women 80.4 cm; 82.7 cm and 85.4 cm; WHR: men 0.92; 0.92 and 0.90; women 0.82; 0.83 and 0.84; C index: men 1.24; 1.24 and 1.24; women 1,20; 1.22 and 1.19; LAP: men 29.81; 32.39 and 33.08; women 22,64; 30.27 and 27.12; VAI: men 1.74; 2.08 and 1.68; women 1.44; 2.16 and 1.65. We chose to use the term "adiposity" instead of obesity for the five indicators, considering that LAP and VAI reflect the function of visceral fat, and not only the accumulation of abdominal fat, such as WC, WHR and C index.^^10^^^,^^^11^^

### Covariates

Ethnicity/skin color was self-attributed and categorized as white, brown and black. The level of schooling was categorized as complete college/university education, complete high school and incomplete and complete elementary school. Smoking was stratified as nonsmokers, ex-smokers, and current smokers.

Weight and height were measured with participants wearing the study clothing, without shoes and accessories. A Toledo scale and a Seca stadiometer were used for the measurements of weight and height, respectively. These variables were used to calculate adiposity indexes.

Blood samples were collected by venipuncture after 12 hours of fasting. Triglyceride and HDL-cholesterol tests were performed by colorimetric enzymatic and homogeneous enzymatic colorimetric methods without precipitation, respectively. LDL-cholesterol levels were obtained using Friedewald's formula. Triglycerides and HDL-cholesterol were used to calculate the LAP and VAI.

Arterial hypertension was defined with a mean systolic blood pressure ≥ 140 mmHg and a mean diastolic ≥ 90 mmHg; or if the individual was undergoing antihypertensive treatment. Blood pressure was measured three times, considering the mean of the last two measurements for calcualtion.^^15^^

### Statistical analysis

A data descriptive analysis was carried out to evaluate the distribution of participants according to the characteristics of interest. Due to the asymmetric distribution of some variables it was decided to show the continuous variables as median and interquartile range. Categorical variables were expressed as absolute and relative frequencies.

The frequency of high CIMT (≥75^th^ percentile) and abdominal adiposity through WC, WHR, C index, LAP and VAI indicators were estimated. Regression coefficients and odds ratios (OR), with their respective 95% confidence intervals, were calculated using linear regression and multivariate logistic analyses, respectively. Regression analyses were used to identify the magnitude of the effect of the abdominal adiposity presence, measured by the indicators in a categorical scale, on the mean of the CIMT in the linear model and on the diagnosis of high CIMT in the logistic analysis.

Due to the asymmetric distribution, CIMT values were transformed into natural logarithm for linear regression. For the logistic regression, the dichotomized CIMT was used in the 75^th^ percentile of the distribution. The main independent variables (abdominal adiposity indicators) were introduced separately in five models for each regression analysis (linear and logistic) by gender. All models were adjusted for age, ethnicity/skin color, level of schooling, smoking status, HDL-cholesterol, LDL-cholesterol, and arterial hypertension, chosen for their proximity to the atherosclerosis condition.^^21^^

An effect modification analysis was performed to test the variables gender and ethnicity/skin color in all proposed models using the maximum likelihood ratio test. No effect modification was detected; however, we maintained the analyses stratified by gender based on theoretical references.^^5^^^,^^^22^^ A diagnostic evaluation of the multiple linear regression models was carried out through graphic analysis of residues, evaluation of influential points and multicollinearity. The Hosmer-Lemeshow test, goodness-of-fit test using the Pearson's residuals and Deviance residues, McFadden's Adjusted R^2^ and ROC curve, were used to diagnose logistic model adjustment. A significance level of 5% was established and the Stata 12 software (Stata Corporation, College Station, Texas, USA) was used for the analyses.

## Results

The sample characteristics are shown in Table 1. Men and women with high CIMT had an older median age (47 and 48 years *versus* 57 years) and a higher frequency of abdominal adiposity (men 71.9% to 78.4%; and women 66% to 73.1%).

**Table 1:** Baseline characteristics, according to the carotid intima-media thickness and gender. ELSA-Brazil, 2008-2010

		Male		Female	
		CIMT < P75	CIMT > P75	CIMT < P75	CIMT > P75
		n = 2,779	n = 958	n = 3,503	n =1,209
Age, median (IQR)		48 (43-54)	57 (51-63)	47 (43-53)	57 (51-62)
**Ethnicity/skin color, n (%)**					
White		1,562 (56.2)	545 (56.8)	2,010 (57.3)	705 (58.3)
Brown		836 (30.0)	266 (27.7)	883 (25.2)	306 (25.3)
Black		381 (13.7)	147 (15.3)	610 (17.4)	198 (16.3)
**Level of schooling, n (%)**					
Complete College/University		1,352 (48.6)	420 (43.8)	1,976 (56.4)	613 (50.7)
Complete High School		1,049 (37.7)	310 (32.3)	1,292 (36.8)	413 (34.1)
Incomplete + complete Elementary School		378 (13.6)	228 (23.8)	235 (6.7)	183 (15.1)
**Smoking status, n (%)**					
Never smoked		1,588 (57.1)	366 (38.2)	2,284 (65.2)	695 (57.4)
Former smoker		811 (29.1)	404 (42.2)	803 (22.9)	334 (27.6)
Current smoker		380 (13.6)	187 (19.5)	416 (11.8)	180 (14.8)
HDL-cholesterol, median (IQR)		49 (43-57)	49 (43-57)	60 (52-71)	59 (51-70)
LDL-cholesterol, median (IQR)		130 (110-152)	138.5 (117-161)	127 (106-149)	140 (119-164)
Arterial hypertension, n (%)		709 (25.5)	499 (52.1)	644 (18.3)	540 (44.7)
Mean BMI (IQR)		26.0 (23.6-28.5)	27.2 (24.6-29.9)	25.3 (22.7-29.5)	27.3 (24.1-30.4)
**Abdominal adiposity, median (IQR)**					
Waist circumference		92.3 (85.5-99.4)	96.6 (89.4-104.1)	83.2 (76.5-91.4)	88.9 (81-97.3)
Waist-to-hip ratio		0.93 (0.88-0.97)	0.96 (0.92-1.00)	0.82 (0.78-0.87)	0.86 (0.81-0.91)
Conicity index		1.26 (1.21-1.30)	1.29 (1.24-1.34)	1.19 (1.14-1.25)	1.23 (1.18-1.29)
Lipid accumulation product		38.8 (22.1-65.3)	51.2(30.4-82.2)	26.48 (15.3-44.4)	39.9 (23.4-63.3)
Visceral adiposity index		2.41 (1.47-3.95)	2.91 (1.74-4.66)	1.62(1.06-2.61)	2.15 (1.37-3.43)
**Abdominal adiposity, n (%)**					
Waist circumference		1,599 (57.5)	690 (72.0)	1,939 (55.3)	884 (73.1)
Waist-to-hip ratio		1,628 (58.5)	751 (78.3)	1,744 (49.7)	847 (70.0)
Conicity index		1,740 (62.6)	738 (77.0)	1,657 (47.3)	798 (66.0)
Lipid accumulation product		1,670 (60.0)	715 (74.6)	1,834 (52.3)	865 (71.5)
Visceral adiposity index		1,774 (63.8)	708 (73.9)	1,733 (49.4)	799 (66.0)

The sum of observations may differ in some variables due to data loss; CIMT: carotid intima-media thickness; P75: 75th percentile; IQR: interquartile range; n (%): number of observations (frequency); BMI: body mass index.

The values of abdominal adiposity indicators were higher in men and in men and women with CIMT > 75^th^ percentile. The men had a median CIMT of 0.59 mm (0.52-0.69), and women of 0.56 mm (0.50-0.64) (data not shown).

In both genders, the adiposity measured by the five indicators was associated with the mean log of CIMT. The C index showed the smallest effect (Table 2).

**Table 2:** Multivariate linear regression analysis between abdominal adiposity, measured by five indicators alone, and CIMT, according to gender. ELSA-Brazil 2008-2010

		Male		Female	
		n = 3,737		n = 4,712	
		β (SE)	95%CI	β (SE)	95%CI
Waist circumference		0.045 (0.006)	0.033;0.058	0.025 (0.004)	0.016;0.035
Waist-to-hip ratio		0.032 (0.006)	0.019;0.045	0.026 (0.004)	0.016;0.035
Conicity index		0.016 (0.006)	0.003;0.029	0.011 (0.004)	0.002;0.020
Lipid accumulation product		0.030 (0.006)	0.016;0.043	0.024 (0.004)	0.014;0.034
Visceral adiposity index		0.022 (0.007)	0.007;0.037	0.020 (0.005)	0.010;0.031

The models were adjusted for age, ethnicity/skin color, level of schooling, smoking status, HDL-cholesterol, LDL-cholesterol and arterial hypertension.

According to the multivariate logistic regression analysis (Table 3), there was an association between the diagnosis of adiposity by WC, WHR, LAP and VAI with CIMT in both genders. The adiposity diagnosed by WC showed a greater effect on CIMT in both genders. According to the diagnostic analyses of the models, there were no assumption violations, indicating the models' adequacy.

**Table 3:** Odds ratio and respective 95% confidence intervals for the association between abdominal adiposity, diagnosed by five indicators alone, with CIMT, according to gender. ELSA-Brazil 2008-2010

		Male		Female
		n = 3,737		n = 4,712
		OR (95%CI)		OR (95%CI)
Waist circumference		1.47 (1.22;1.77)		1.38 (1.17;1.64)
Waist-to-hip ratio		1.37 (1.12;1.67)		1.33 (1.13;1.57)
Conicity index		1.02 (0.83;1.24)		1.12 (0.95;1.32)
Lipid accumulation product		1.39 (1.13;1.69)		1.28 (1.08;1.53)
Visceral adiposity index		1.42 (1.13;1.77)		1.31 (1.08;1.59)

The models were adjusted for age, ethnicity/skin color, level of schooling, smoking status, HDL-cholesterol, LDL-cholesterol and arterial hypertension.

## Discussion

Using data from the ELSA-Brasil study, associations were observed between abdominal adiposity measurements and CIMT, a noninvasive marker of subclinical atherosclerosis capable of predicting cardiovascular disease.^^23^^ It has been documented, in a study carried out in Southeast Brazil, the definition of CIMT as the thickening of the intima-media complex starting from 1.0mm.^^24^^ Considering this value, in our study, the presence of abdominal adiposity diagnosed by WC, WHR, LAP and VAI showed an important effect, with a variation of 0.02 mm to 0.04 mm in the log of CIMT in both genders. Polack et al.,^^23^^ using data from the Framingham offspring cohort study, found that an annual change in CIMT > 0.02 mm was associated with a more than two-fold risk of cerebrovascular accident.^^23^^

Few studies have compared different indicators of adiposity with CIMT, and the present study is the first one that separately investigated the contribution of different indicators of abdominal adiposity. Previous studies also carried out with ELSA-Brazil data also evaluated the association between traditional risk factors and CIMT.^^25^^^,^^^26^^ WC, WHR, waist-to-height ratio (WHtR) and neck circumference (NC) were included in the analysis. The latter indicator had the strongest association with CIMT. The authors suggest that the local effect produced by neck fat acts directly on the carotid arteries.^^25^^^,^^^26^^ Our study did not include neck circumference; however, the measures used in the study are relatively simple and reflect important information about the risk of developing cardiovascular diseases, at individual and population levels.^^27^^

Most studies that evaluated the association between abdominal adiposity and CIMT used visceral fat measured by imaging tests. In these studies, visceral fat was strongly associated with CIMT,^^28^^^,^^^29^^ but the comparison with these findings is limited by the different methods used to identify abdominal and visceral fat. The association between abdominal adiposity and subclinical atherosclerosis is possibly related to the visceral component of abdominal fat. The indicators evaluated in the present study are indirect measures of this component, but they show good correlation with visceral fat and are accessible to the overall population.^^27^^

The WC was the indicator most strongly associated with CIMT. Similar to our data, other studies have also found an association between WC and CIMT in healthy 45- to 65-year-old Dutch adults, hospitalized Irish adults, and hospitalized subjects aged 21-83 years in China.^^7^^^,^^^30^^^,^^^31^^ WC is described as an indicator of abdominal adiposity with a greater capacity to predict metabolic alterations and cardiovascular diseases, being one of the measures that most closely approximates to visceral fat measured by imaging tests.^^27^^

In this study, WHR also showed an important association with CIMT between men and women. Large epidemiological studies have described the strongest associations not only between adiposity diagnosed by WHR and CIMT, but also with the prevalence of myocardial infarction, incidence of coronary artery disease, high coronary risk and coronary events.^^6^^^,^^^32^^^,^^^33^^ However , evidence shows that the gluteofemural region consists mainly of subcutaneous adipose tissue. This tissue does not seem to play an important role in the pathogenesis of cardiovascular disease. By including hip measurement, WHR reflects the effect of total adiposity as a risk factor for atherosclerosis and other cardiovascular outcomes.^^32^^ Thus, WHR can be useful as a simple and consistent indicator by reflecting the combination of total and abdominal adiposity.

The C index was the indicator that showed the lowest effect of abdominal adiposity on the CIMT in this study. No studies were found that investigated this indicator in relation to subclinical atherosclerosis. Previous publications have observed the association of this indicator with high coronary risk in Brazilians from the Northeast region^^34^^ and metabolic alterations in Indian civil servants.^^35^^ Although the C index is not a new indicator, it remains little explored and there is no consensus on ideal cutoff points for the Brazilian population. As it considers weight and height, similar to the WHR, it may be useful to demonstrate the combination of total and abdominal adiposities on cardiovascular outcomes. One hypothesis for the absence of association in this study is the large percentage of participants of white ethnicity/skin color, since the performance of this indicator as a discriminator of coronary risk works better in black populations.^^34^^

VAI is an indicator originally proposed to identify the distribution and function of adipose tissue, indirectly expressing cardiovascular risk. Due to the inclusion of physical and metabolic parameters (anthropometric measures and biochemical tests), this indicator may reflect the altered production of adipocytokines, increase in lipolysis and free fatty acids in plasma.^^10^^

Evidence indicates that VAI was independently associated with cardiovascular (OR = 2.45, 95%CI: 1.52, 3.95) and cerebrovascular events (OR = 1.63, 95%CI: 1.06, 2.50) in healthy and non-obese Italians.^^10^^ The only study found that evaluated the association between VAI and a subclinical measure of atherosclerosis - the CAC - coronary artery calcium score - was carried out with 33,468 Koreans with a mean age of 42 years. Similar to the present findings, but with a lower magnitude of association, the highest chance of having subclinical atherosclerosis (OR = 1.26, 95%CI: 1.14, 1.38) was shown in individuals with the highest tertile of VAI.^^9^^ It was found in the current study that the chance of men and women with abdominal adiposity assessed by VAI of having high CIMT was 42% and 31%, respectively. This difference between the studies was possibly observed due to the characteristics of the investigated populations (healthy participants *versus* patients from a Korean university hospital).^^9^^

Similar to VAI, the LAP showed an association between the presence of abdominal adiposity and CIMT. No previous evidence was found on the association between LAP and subclinical atherosclerosis. The LAP was developed to reflect combined metabolic and physical alterations, using WC and triglycerides. Therefore, it measures lipid overaccumulation and stands out as a cardiovascular risk factor in adults. This indicator has been investigated in the context of metabolic and cardiovascular diseases and mortality. An American cohort study with approximately 5,000 subjects treated at a cardiologic clinic between 1995-2006 showed an association between LAP and cardiovascular mortality (HR: 1.52 95%CI: 1.27, 1.82), adjusted for age, gender, smoking, diabetes, blood pressure, LDL-cholesterol and HDL-cholesterol.^^36^^

However, more studies are needed, especially in Brazil, to broaden the knowledge of less popular indicators such as VAI and LAP. Evidence suggests that information not only on the fatty tissue accumulated in the abdominal region is provided through LAP and VAI, but also on fat deposition in areas such as the liver, muscle, heart and arteries. This lipid overaccumulation causes changes in intracellular metabolism and contributes to the occurrence of cardiovascular disease, including atherogenesis and death.^^19^^

In the present study the associations between adiposity measures and CIMT were more significant for men than for women. Women have more total body fat (and subcutaneous), often in the legs and buttocks and, especially, before menopause. Men tend to accumulate fat in the abdominal region throughout life, so they are at higher risk for developing cardiovascular outcomes,^^22^^ including atherosclerosis.

Evidence shows differences in the progression of CIMT and adiposity due to the ethnicity/ skin color.^^37^^ The cut-off points used in this study incorporated the differences between gender and ethnicity/skin color^^20^^ and, perhaps because of that, no effect modification was detected.

Through the coefficients of determination (R^2^), the linear regression model variables, including each indicator alone, explained approximately 30% of the total CIMT variability. In our study, the models were adjusted for age, ethnicity/skin color, level of schooling, smoking, HDL-cholesterol, LDL-cholesterol and arterial hypertension. The study carried out by Santos et al.,^^25^^ using the ELSA-Brazil sample, found coefficients of determination (R^2^) close to 40% when investigating the association of risk factors with CIMT through the variables: blood pressure, glucose metabolism, lipid profile and adiposity (body mass index, WC, hip circumference, WHR, waist-to-height ratio, neck circumference). It is noteworthy that, in addition to adiposity patterns, geographic, genetic, environmental and behavioral characteristics are also associated with the occurrence of atherosclerosis.

The 75^th^ percentile of the distribution was used to categorize CIMT in the logistic regression analysis. Other values for this classification might have yielded more consistent results. However, studies show subjects with CIMT values above the 75^th^ percentile with a higher risk of developing cardiovascular disorders.^^17^^^,^^^38^^ It is known that the atheroma plaques may be more representative of atherosclerosis than CIMT.^^39^^ However, our population is relatively young, and when CIMT was dichotomized at 1.5 mm, a proposed classification for atheroma plaque according to the international consensus,^^5^^ it showed a low frequency of participants with this condition (4% in men and 2% in women) (data not shown).

The use of a stringent protocol for image acquisition and quality control provided reliable and accurate data of CIMT measurements in this study. To reduce the influence of the evaluator, the reading of all images was centralized, and the automated measurements were performed by software. Although we did not adjust the models by body mass index, we excluded subjects with class III obesity and those who underwent bariatric surgery from the analysis, aiming to filter the effect of abdominal adiposity without influence of excessive total body fat.

This study has limitations. Data on menopause were not considered. When women reach menopause they lose the protection provided by the hormone estrogen and, as they get older, there is a greater accumulation of abdominal fat, as well as an increase in the occurrence of cardiovascular problems.^^22^^ The literature is clear about the effect of age on atherosclerosis.^^5^^ Although the analyzes were adjusted for age in this article, it did not allow the observation of the effect of adiposity on CIMT at different age groups. It is not possible to affirm causality due to the cross-sectional design of this study; however, it seems unlikely that arterial thickening occurs before the high accumulation of abdominal fat. ELSA-Brazil is an occupational cohort and generalizations for the Brazilian population are limited, despite similarities in the prevalence indicators observed in ELSA-Brasil and VIGITEL studies.^^40^^

## Conclusion

The observed results reinforce the importance of abdominal adiposity for the condition of subclinical atherosclerosis. Abdominal adiposity, identified through WC, WHR, LAP and VAI, was associated with CIMT in both genders, with the traditional WC anthropometric indicator standing out. WC, when compared to the other indicators, and men, when compared to women, showed the most significant effects.
